# Detection of Alpha-1 Antitrypsin Levels in Chronic Obstructive Pulmonary Disease in Respiratory Clinics in Spain: Results of the EPOCONSUL 2021 Audit

**DOI:** 10.3390/jcm13040955

**Published:** 2024-02-07

**Authors:** Myriam Calle Rubio, Marc Miravitlles, José Luis López-Campos, Juan J. Soler-Cataluña, Bernardino Alcazar Navarrete, Manuel E. Fuentes-Ferrer, Juan Luis Rodriguez Hermosa

**Affiliations:** 1 Pulmonology Department, Hospital Clínico San Carlos, Instituto de Investigación Sanitaria del Hospital Clínico San Carlos (IdISSC), 28040 Madrid, Spain; jlrodr01@ucm.es; 2 Department of Medicine, School of Medicine, Universidad Complutense de Madrid, 28040 Madrid, Spain; 3 Pulmonary Department, Hospital Universitari Vall d’Hebron, Vall d’Hebron Institut de Recerca (VHIR), Vall d’Hebron Barcelona Hospital Campus, 08035 Barcelona, Spain; marcm@separ.es; 4 Centro de Investigación Biomédica en Red de Enfermedades Respiratorias (CIBERES), Instituto de Salud Carlos III, 28029 Madrid, Spain; lcampos@separ.es; 5 Respiratory Disease Medical-Surgical Unit, Instituto de Biomedicina de Sevilla (IBiS), Hospital Universitario Virgen del Rocío/Universidad de Sevilla, 41009 Sevilla, Spain; 6 Pulmonology Department, Hospital Arnau de Vilanova-Lliria, 46015 Valencia, Spain; jjsoler@telefonica.net; 7 Medicine Department, Valencia University, 46010 Valencia, Spain; 8 Pulmonary Department, Hospital Universitario Virgen de las Nieves, Instituto Biosanitario de Granada, 18014 Granada, Spain; balcazarnavarrete@gmail.com; 9 Unidad de Investigación, Hospital Universitario Nuestra Señora de Candelaria, 38010 Santa Cruz de Tenerife, Spain; mfuentesferrer@gmail.com

**Keywords:** α1-antitrypsin deficiency, diagnosis, chronic obstructive pulmonary disease, clinical audit, outpatient respiratory clinics

## Abstract

Background: Alpha-1 antitrypsin deficiency (AATD) is an underdiagnosed condition despite being one of the most common inherited disorders in adults that is associated with an increased risk of developing chronic obstructive pulmonary disease (COPD). The aim was to evaluate the frequency of performing AAT levels and associated factors in COPD patients in an audit conducted in 2021–2022, as well as to compare with a previous audit conducted in 2014–2015. Methods: EPOCONSUL 2021 is a cross-sectional audit that evaluated the outpatient care provided to COPD patients in respiratory clinics in Spain based on available data from medical registries. Results: 4225 patients with a diagnosis of COPD from 45 centers were audited in 2021. A total of 1670 (39.5%) patients underwent AAT determination. Being treated at a specialized COPD outpatient clinic (OR 1.88, *p* = 0.007), age ≤ 55 years old (OR 1.84, *p* = 0.007) and a FEV1 < 50% (OR 1.86, *p* < 0.001) were associated with a higher likelihood of being tested for AAT, while Charlson index ≥ 3 (OR 0.63, *p* < 0.001) and genotyping of AATD availability (OR 0.42, *p* < 0.001) showed a statistically significant negative association. The analysis of cases included in respiratory units that participated in both audits showed an increase in the proportion of cases with AAT serum level testing available (adjusted OR 2.81, *p* < 0.001). The percentage of individuals with serum AAT levels < 60 mg/dL (a severe AATD) was 4%. Conclusions: Our analysis identifies significant improvements in adherence to the recommendation to test AAT levels in COPD patients, performed in 4 out of 10 patients, being more likely at younger ages and with higher COPD severity, and with a detection of severe AATD of 4% among those tested, suggesting that clinicians still perform AAT testing in COPD patients selectively. Therefore, efforts are still needed to optimize AATD screening and establish new early detection strategies to reduce morbidity and mortality in these patients.

## 1. Introduction

Alpha-1 antitrypsin deficiency (AATD) is one of the most common hereditary disorders in adults [1]. It is characterized by abnormally reduced serum alpha-1 antitrypsin (AAT) levels and associated with increased risk for the development of early-onset pulmonary emphysema and liver disease [2]. It has been suggested that 1–2% of subjects with chronic obstructive pulmonary disease (COPD) have AATD [3] and that around 1/800 patients with COPD in Europe have severe AATD [4]. Despite its considerable prevalence, healthcare providers still fail to search for AATD in subjects with pulmonary diseases, and this condition remains largely underdiagnosed [5,6,7]. Diagnosis is made by the demonstration of reduced blood levels of AAT. Recommendations of healthcare institutions such as the World Health Organization (WHO) [8], the Spanish National Guidelines for Chronic Obstructive Pulmonary Disease (GesEPOC) [9] and the American and European Thoracic/Respiratory Societies (ATS/ERS) [10,11] indicate that all COPD patients should be tested for AATD at least once during their lifetime. However, the real-life implementation of these clinical practice guidelines (CPG) is low [12,13,14,15]. 

Clinical audits have emerged as a tool to assess healthcare and provide healthcare professionals with information they can use to improve clinical care provided to patients and clinical outcomes. In this context, Spain used an auditing process that evaluated outpatient care provided to patients with COPD in respiratory clinics in Spain, called EPOCONSUL [15,16], which resulted in the completion of two clinical audits based on available data from medical records. The first of these audits was performed between 1 May 2014, and 1 May 2015, and the second between 15 April 2021, and 31 January 2022. 

In the present study, we evaluated the results of two consecutive clinical audits performed in Spain to assess the outpatient care provided to COPD patients. This analysis evaluates the frequency of AAT levels and associated factors in the 2021−2022 audit and compares it with data from the 2014−2015 audit conducted in the same centers. It also analyzes the frequency and clinical characteristics of COPD patients by AAT level. The aim is to provide health professionals with information that they can use to assess and adjust their performance.

## 2. Materials and Methods

The methodology of the 2021 EPOCONSUL audit is similar to that of the 2015 EPOCONSUL audit, which has been reported in previous publications [15,16]. The COPD audit promoted by the Spanish Society of Pneumology and Thoracic Surgery (SEPAR) was designed to evaluate clinical practice as well as clinical and organizational factors related to managing patients with COPD across Spain. It was designed as an observational non-interventional cross-sectional study. The SEPAR sent an official invitation to participate in the study to all the respiratory units in Spain with outpatient respiratory clinics according to the Registry of the Ministry of Health and to the register of members of the SEPAR. Participating investigators in the 2021 EPOCONSUL are included in Supplementary Table S1. Recruitment was intermittent; every month, each investigator recruited the clinical records of the first 10 patients identified as being diagnosed with COPD that were seen in the outpatient respiratory clinic. Subsequently, the patients identified were reevaluated to determine whether they met the inclusion/exclusion criteria described in Supplementary Table S2. The information collected was historical in nature for the clinical data from the last visit pre-pandemic (performed before March 2020) and previous visits; the information about hospital resources was concurrent and is described in Supplementary Table S3.

Based on the serum AAT levels, individuals were classified as follows: no deficiency, AAT ≥ 116 mg/dL; intermediate deficiency, AAT between 60 mg/dL and 116 mg/dL; and severe deficiency, AAT < 60 mg/dL [17].

The protocol was approved by the Ethics Committee of the Hospital Clínico San Carlos (Madrid, Spain; internal code 20/722-E, approval 11/2020). Additionally, according to current research laws in Spain, the ethics committee at each participating hospital evaluated and agreed to the study protocol. The need for informed consent was waived because this was a clinical audit, in addition to the non-interventional nature of the study, the anonymization of data and the blind evaluation of the clinical performance. This circumstance was clearly explained in the protocol, and the ethics committees approved this procedure. To avoid modifications to the usual clinical practice and preserve the blinding of the clinical performance evaluation, the medical staff responsible for the outpatient respiratory clinic were not informed about the audit. Data were entered remotely at each participating location to a centrally controlled server.

### Statistical Analysis

Qualitative variables were summarized by their frequency distribution and quantitative variables by their mean and standard deviation (SD). The association between each independent variable (patient characteristics and hospital resources) and the dependent variable AAT level determination were assessed by calculating the crude odds ratio (OR) via logistic regression with cluster robust standard errors to account for patients being tested within hospitals. A multivariable logistic model was fitted in order to evaluate the independent effect of the selected variables. Candidate predictors with a value of *p* < 0.10 in the bivariate analysis were accepted for inclusion in the multivariate analysis.

To compare the determination of AAT levels between the two audits, a logistic regression with cluster robust standard errors was fitted including only patients from hospitals that participated in both audits. The adjustment variables of the model were those factors that were related to the determination of AAT in both the first and second audit and were collected in a homogeneous way.

AAT levels were grouped into three categories (<60 mg/dL, ≥60–<116 mg/dL, and ≥116 mg/dL). Comparisons between the ≥116 group and the other groups were made by logistic regression with cluster robust standard errors. Statistical significance was assumed as *p*< 0.05. All analyses were performed using Stata software version 16 (StataCorp LLC, CollegeStation, TX, USA).

## 3. Results

A total of 4225 patients with a diagnosis of COPD from 45 centers were audited. Of the analyzed cohort, only 1670 (39.5%) patients underwent AAT determination. 

### 3.1. Characteristics of the Participating Hospitals and Resources of the Respiratory Units and Their Association with AAT Testing

Most participating centers were university hospitals (85%) and had a complexity level III (77.4%). There were 64.6% centers with a specialized COPD outpatient clinic, and the availability of AAT genetic testing was 88.8%. The center-level variables were not associated with AAT testing, except the availability of genotyping of AAT (OR 0.58, *p* = 0.030) and being treated at a specialized COPD outpatient clinic (OR 1.63, *p* = 0.032). Table 1 describes the logistic regression bivariate analysis with the variables related to centers and the association with AAT testing.

### 3.2. Clinical Characteristics of the Audited Cases and Their Association with AAT Testing 

Table 2 describes the characteristics of the evaluated patients in the 2021 audit and the patient-level variables that were associated with AAT level determination in logistic regression bivariate analysis. In the adjusted model, summarized in Table 3, being treated at a specialized COPD outpatient clinic (OR 1.88, *p* = 0.007), age ≤ 55 years old (OR 1.84, *p* = 0.007) and a FEV1(% predicted) < 50% (OR 1.86, *p* < 0.001) were associated with a higher likelihood of being tested for AAT, while Charlson index ≥ 3 (OR 0.63, *p* < 0.001) and genotyping of AATD availability (OR 0.42, *p* < 0.001) showed a statistically significant negative association. 

### 3.3. Variations in Testing AAT between the 2 Audits 

**Figure 1:**
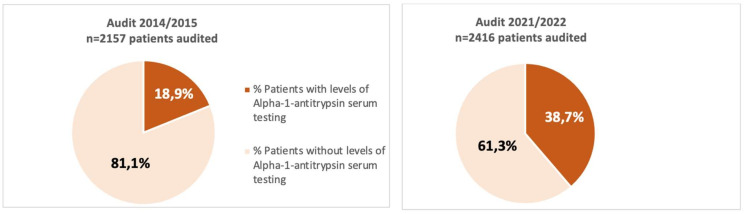
Distribution of patients according to availability of determination of alpha-1 antitrypsin levels in serum in 2014/2015 and 2021/2022 in the centers that participated in both audits.

A total of 25 hospitals participated in both audits. The analysis of cases included in respiratory units that participated in both audits showed an increase in the proportion of cases with AAT serum level testing available (audit 1: 18.9%; audit 2: 38.7%, *p* < 0.001), presented in Figure 1. In the adjusted model, the analysis showed an increase in the percentage of requests adjusting for factors influencing the request between the two audits, (OR 2.81, *p* < 0.001), summarized in Table 4, which shows some characteristics of interest in relation to testing AAT of patients and centers that participated in both audits.

### 3.4. Clinical Characteristics of Patients According to AAT Levels and Procedures Conducted for COPD Evaluation 

**Figure 2:**
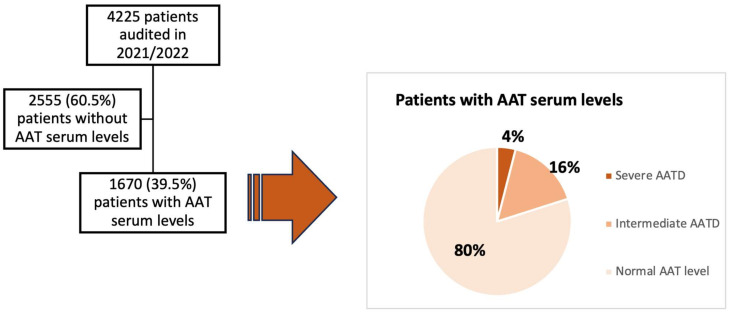
Distribution of patients according to availability of AAT level determination and based on the AAT levels in patients audited in 2021/2022. Severe AATD: serum AAT levels < 60 mg/dL; Intermediate AATD: serum AAT levels between 60 mg/dL and 116 mg/dL; Normal AAT level: serum AAT levels > 116 mg/dL.

In total, 331 (20%) of the 1651 patients tested had serum AAT levels < 116 mg/dL. Of them, 265 (16) patients had serum AAT levels between 60 mg/dL and 116 mg/dL (might be considered an intermediate AATD) with a mean plasma AAT level of 98.9 (SD 14.2) mg/dL, while 66 (4% of those tested) patients had serum AAT levels < 60 mg/dL (a severe AATD) with a mean plasma AAT level of 19.3 (SD 14.6) mg/dL (Figure 2). Patients with severe AATD had more dyspnea (MRC-m) ≥ 2 (OR 2.56, *p* < 0.001), were more likely to have phenotype of exacerbator chronic bronchitis (OR 3.34, *p* = 0.023) and were less likely to be active smokers (OR 0.58, *p* = 0.023) than individuals with normal AAT levels ≥116 mg/dL. During follow-up, the chest CT scan was more frequently carried out in patients with severe AATD (OR 13.6, *p* = 0.026), as summarized in Table 5.

## 4. Discussion

This study provides data about the changes that have occurred in the request of AAT levels in patients with COPD treated in outpatient respiratory clinics in Spain and analyzes the factors associated with requesting this test. 

Our analysis showed increased adherence to the good clinical practice recommendation that all COPD patients should be screened for AATD at least once in their lifetime. AAT levels were measured in almost four out of ten COPD patients in pulmonology clinics in Spain in the 2021 audit (38.7%) compared to almost two out of ten (18.9%) in the 2015 audit. 

In Spain, a cross-cutting audit process was conducted with a 5-year interval between the two audits, during which numerous communications of results, presentation meetings and discussions of the findings of the first audit were carried out with the aim of improving the quality of care. In addition, during this period between the two audits, several updates to good clinical practice guidelines [9] and strategic documents on COPD management [10,11] were published, with an extensive dissemination plan for good clinical practice recommendations, aiming to improve the quality of care, with special emphasis on the most deficient areas based on the results of the first audit, such as the request for AAT levels in COPD.

AATD is a highly underdiagnosed condition [5]. Early diagnosis is important to allow physicians to take preventive measures, such as actively promoting smoking cessation and awareness to avoid exposure to respiratory pollutants and initiate appropriate treatment when necessary. Patients with severe AATD may benefit from augmented AAT infusion therapy that protects the lung against the action of neutrophil elastase and, thus, slows the progression of emphysema [18].

Underdiagnosis of AATD remains a reality, as demonstrated by the results of this analysis. Although the frequency of testing has increased compared to the 2015 audit, 60% of COPD patients cared for in pulmonology outpatient clinics still do not have an AAT blood test despite prolonged follow-up in 10 specialized clinics of university hospital centers with a high level of complexity. Although the diagnosis of AATD is easy and cheap, studies have indicated that AATD is underdiagnosed and delay in its diagnosis is frequent. Patients with AATD typically experience a delay of 5 to 8 years between the onset of symptoms and confirmation of AATD [19,20,21]. These results point to the lack of awareness and understanding of AATD among pulmonologists, who forget to request serum AAT levels in most of their COPD patients, as a determining factor in this underdiagnosis. These data reflect a failure to follow CPG recommendations to detect cases of AATD in risk groups, such as in patients diagnosed with COPD. Studies on the level of training for AATD have shown poor knowledge among doctors, even those interested in respiratory diseases, and few respiratory specialists report routinely performing AAT tests on all their COPD patients [22,23]. With fewer than 10% of affected individuals being clinically diagnosed [5,24], improving the use of this targeted detection starts by raising physician awareness.

With regard to the factors associated with determining testing for AAT, in our study, no association was found between resources in the pulmonology units and a higher probability of determining AAT levels; however, it is important to note that only the availability of genetic testing was associated with a lower likelihood of determining AAT levels—a result that may reflect a different clinical practice in the diagnostic algorithm of AATD, where according to current recommendations, quantitative measurement of serum AAT in patients with stable COPD is the first test to be performed in the diagnostic process [25]. Although there is no universally accepted laboratory algorithm for the diagnosis of AATD, when serum AAT concentration is below the reference range, the study should be completed with phenotyping and/or genotyping [17,26]. In Spain, a new diagnostic tool has been available since October 2018, the AAT genotyping test from Progenika, which allows simultaneous analysis of the 14 most frequent deficient mutations based on multiplex technology with the Luminex 200 Instrument System [27]. This is an easily accessible procedure that has emerged to reduce the underdiagnosis of AATD, and may in-turn be contributing to a change in the diagnostic algorithm, taking precedence over the determination of AAT levels in places where it is available. 

Moreover, being treated in a specialized COPD clinic was associated with a higher likelihood of determining AAT levels in blood in our study. A result that could be related to increased interest and knowledge of professionals treating patients in 11 specialized clinics. Physicians’ awareness, knowledge and attitude towards AATD may be key elements in explaining the failure (or success) to screen their COPD patients for AATD and the delay in diagnosis. Early diagnosis is the key to improving the prognosis of AATD-related disease because it leads to early treatment and prevention of risk factors, and eventually to improved patient outcomes [19]. Cigarette smoking has an adverse effect on the course of lung disease and is by far the single most important risk factor for the development of rapidly progressive COPD in patients with AATD [28]. Timely detection can allow for appropriate counseling on lifestyle modifications (e.g., smoking cessation and vaccination) and augmentation therapy for candidates patients. Accurately diagnosing patients with AATD could also enable genetic counseling and targeted screening to identify at-risk family members.

With respect to patient-level variables, in our analysis, the determination of AAT levels was associated with demographic (age ≤ 55 years) and clinical factors (lower FEV1 % pred and lower comorbidity burden). Young age is a trait that has classically been attributed to COPD due to AATD, which would point to a clinical practice guided by clinical suspicion. However, different series have described that AATD is detected in different clinical forms of COPD [29,30,31], so there are no definitive clinical features on which to base suspicion of AATD.

Although the prevalence studies of AATD have estimated that approximately 1/3500 and 1/6000 individuals of European descent may be affected by severe AATD in its homozygous PI*ZZ form [4], and although the guidelines recommend testing AAT levels in target populations, such as individuals with COPD [8,9,10,11], AATD in COPD remains underdiagnosed [5,6,16]. It has been estimated that around 1/800 patients with COPD in Europe have severe AATD [4,32]. Published studies of screening programs in COPD patients have reported approximately 1–2% of cases with severe AATD [33,34,35]. 

In our analysis, we found 4% of cases of severe AATD among those tested. This detection rate could point to a more selective search strategy, guided by the presence of clinical features that might suggest a higher likelihood of AATD such as younger age and greater severity of COPD, which in our analysis were identified as factors associated with the determination of AAT levels. Analysis of the clinical characteristics of COPD patients according to whether or not they had AATD showed no significant differences in most variables. Patients with severe AATD versus COPD patients without AATD were more symptomatic, with a higher history of severe exacerbations and comorbidity burden, and a lower frequency of current smoking. Previous studies have shown that the clinical manifestations of COPD associated with AATD are highly variable and largely dependent on tobacco use, the presence of bronchial hyperresponsiveness and recurrent respiratory infections [36]. Clinical data indicate that the severity of the symptoms found in AATD patients is highly variable and neither AAT serum levels nor phenotype are sufficient to identify which patients develop severe COPD [37]. This may favor the fact that clinicians may forget to ask for serum AAT concentrations in many COPD patients. Good clinical practice guidelines [8,9,10,11] stating testing for AATD should be conducted for all COPD patients. Traditionally, however, testing has been carried out on individuals presenting with early-onset emphysema. This diagnostic pathway has resulted in AATD being a condition which is likely both underdiagnosed and diagnosed too late for some therapeutic interventions to have maximum effect. A factor to consider is the time taken for a diagnosis to be confirmed. A delay in diagnosis, which is associated with the deterioration of the patient’s health, has been estimated as up to 7 years [38]. Screening on the basis of clinical data is not sufficient to detect AATD cases. A proactive approach is needed to detect them through a systematic screening program. A number of countries have established screening programs in the adult population with remarkable results [39,40]. Thus, both selective screening of at-risk groups and family screening have the potential to be powerful tools to improve the rate of AATD diagnosis in areas with high AATD prevalence.

In our study, we also found no differences in the frequency of diagnostic procedures performed in the follow-up of COPD in patients with and without AATD, except for a higher frequency of thoracic computed tomography (CT) in patients with severe AATD. In our analysis, more than three quarters of COPD patients, irrespective of the presence of AATD, had undergone diffusing capacity of the lung for carbon monoxide lung test and thoracic CT. This is in line with the results of a survey on the management of AATD conducted by AATD experts where diffusion capacity was considered the most useful measure for monitoring AATD in routine clinical practice [41].

Our study has some limitations that should be considered, such as the fact that the centers were not randomly selected and were chosen based on having participated previously in COPD clinical audits and their interest in the study. Additionally, as in all clinical audits, despite inclusion methodology and periodic supervision of the database, some of the values were not included as they were not available. We must also mention the possible limitation in the diagnosis of the deficiency of AAT only based on the concentration of protein in blood. We must keep in mind that as an acute phase reactant, infectious or inflammatory processes may alter AAT determination [42]; thus, normal or high values may be detected in individuals with an intermediate deficiency. However, despite these limitations, the sample included is likely representative of medical attention for patients with COPD in outpatient respiratory clinics in Spain.

## 5. Conclusions

Our data show a higher frequency of AAT determination in COPD patients, performed in four out of ten patients, although its determination is still associated with clinical characteristics such as younger age and higher severity of COPD, or being seen in a specialized COPD clinic, suggesting that clinicians still screen for AATD in COPD in a selective way. Screening for AATD in all COPD patients is key to improving underdiagnosis and achieving early diagnosis. Therefore, to improve the detection of AATD in COPD, it remains necessary to raise awareness and train physicians in areas where the disease is most likely to occur: pulmonology, hepatology, pediatrics and primary care physicians, as well as to establish new early detection strategies. 

## Figures and Tables

**Table 1:** Characteristics of the participating hospitals, resources of the respiratory units and association with levels of AAT available (logistic regression bivariate analysis).

	All Patients (*n* = 4.225)	AAT Levels Test *n* = 1670 (39.5%)	AAT Levels Not Tested *n* = 2555 (60.5%)	OR (95%CI)	*p*
Hospital complexity level, n (%)					
Level II (reference)				1	
Level III	3271 (77.4)	1255 (75.1)	2016 (78.9)	0.80 (0.43–1.49)	0.499
University hospital, n (%)					
No (reference)				1	
Yes	3593 (85)	1412 (84.6)	2181 (85.4)	0.93 (0.47–1.84)	0.853
Minutes at outpatient respiratory visit available, n (%)					
<15 min (reference)				1	
≥15 min	2258 (53.4)	979 (58.6)	1279 (50.1)	1.41 (0.85–2.32)	0.173
Genotyping of AATD availability, n (%)					
Not available (reference)				1	
Yes	3750 (88.8)	1426 (85.4)	2324 (91)	0.58 80.35–0.94)	0.030
Specialized COPD outpatient clinic available, n (%)					
Not available (reference)				1	
Yes	2729 (64.6)	1076 (64.4)	1653 (64.7)	0.98 (0.58–1.66)	0.965
Outpatient respiratory nursing clinic availability, n (%)					
Not (reference)				1	
Yes	2373 (56.2)	969 (58)	1404 (55)	1.13 (0.67–1.88)	0.632
Written COPD protocol available, n (%)					
Not available (reference)				1	
Yes	1747 (41.3)	761 (45.6)	986 (38.6)	1.33 (0.78–2.25)	0.286
Attended in specialized COPD outpatient clinic, n (%)					
Not available (reference)				1	
Yes	1620 (38.5)	757 (45.5)	863 (33.9)	1.63 (1.04–2.55)	0.032

Data are represented as absolute (relative) frequencies. Odd Ratio (OR), 95% confidence interval and *p* value obtained via logistic regression with cluster robust standard errors.

**Table 2:** Characteristics of the patient and association with levels of AAT available (logistic regression bivariate analysis).

	All Patients (*n* = 4.225)	Patients with Levels of AAT *n* = 1670 (39.5%)	Patients without Levels of AAT *n* = 2555 (60.5%)	OR (95%CI)	*p*
Sex, n (%)					
Male (reference)				1	
Female	1152 (27.2)	498 (29.8)	654 (25.5)	1.23 (1.04–1.47)	0.016
Age (years), m (SD)	70.0 (9.3)	67.9 (8.9)	71.3 (9.2)	0.96 (0.94–0.97)	<0.001
>55, (n, %) (reference)				1	
≤55, (n, %)	268 (6.3)	150 (9)	118 (4.6)	2.03 (1.36–3.04)	0.001
IPA (×10 Pack-years), m (SD)	49.6 (24.0)	48.9 (23.1)	50.1 (24.6)	0.99 (0.99–1.00)	0.368
Active smokers, n (%)					
No (reference)				1	
Yes	1053 (24.9)	439 (26.3)	614 (24)	1.12 (0.91–1.38)	0.225
BMI kg/m2, m (SD)	27.8 (5.6)	27.1 (5.6)	28.3 (5.6)	0.96 (0.95–0.97)	0.001
>21 (n, %) (reference)				1	
≤21 (n, %)	364 (9.1)	172 (10.6)	192 (8.1)	1.33 (1.02–1.74)	0.031
Charlson index, n (%)					
<3 (reference)				1	
≥3	1185 (28.1)	388 (23.3)	797 (31.2)	0.66 (0.54–0.81)	<0.001
Dyspnea (MRC-m), n (%)					
<2 (reference)				1	
≥2	1787 (56)	746 (54.8)	1041 (56.9)	0.91 (0.70–1.189	0.512
Chronic bronchitis criteria, n (%)					
Not (reference)				1	
Yes	1298 (30.7)	552 (33.1)	746 (29.2)	1.19 (0.89–1.599	0.217
History of asthma, n (%)					
Not (reference)					
Yes	411 (9.7)	211 (12.6)	200 (7.8)	1.70 (1.11–2.60)	0.014
FEV1 (%predicted post-BD), m (SD) ≥ 80,	53.1 (18.5)	50.7 (18.1)	54.7 (18.6)	0.98 (0.98–0.99).	<0.001
n (%) (reference)	346 (8.2)	96 (5.8)	250 (9.8)	1	
50–79%, n (%)	1952 (46.3)	727 (43.6)	1225 (48)	1.54 (1.13–2.10)	0.006
<50%, n (%)	1992 (45.5)	846 (50.7)	1076 (42.2)	2.04 (1.48–2.82)	<0.001
GesEPOC Phenotype, n (%)					
Non-exacerbator (reference)	1226 (46.2)	515 (43.3)	711 (48.5)	1	
Exacerbator with chronic bronchitis	446 (16.8)	190 (16)	256 (17.5)	1.02 (0.71−1.8)	0.893
Exacerbator with emphysema	570 (21.5)	279 (23.4)	291 (19.8)	1.32 (0.94−1.8)	0.101
Asthma-COPD	414 (15.6)	206 (17.3)	208 (14.2)	1.36 (0.93−1.9)	0.103
Number of hospital admissions in the last year, n (%)					
<1 (reference)				1	
≥1	692 (16.4)	316 (18.9)	376 (14.7)	1.35 (1.03−1.76)	0.025
Chronic colonization, n (%)					
Not (reference)				1	
Yes	494 (11.7)	227 (13.6)	267 (10.5)	1.34 (0.83−2.17)	0.219
Triple therapy, n (%)					
Not (reference)				1	
Yes	2034 (50.3)	870 (54.3)	1164 (47.7)	1.30 (1.07−1.58)	0.007
Long-term oxygen therapy, n (%)					
Not (reference)				1	
Yes	1059 (25.1)	408 (24.4)	651 (25.5)	0.94 (0.76−1.16)	0.601
Home ventilation, n (%)					
Not (reference)				1	
Yes	362 (8.6)	125 (7.5)	237 (9.3)	0.79 (0.56−1.11)	0.180
Respiratory care follow-up (years), (median, IQR)	5.8 (3.6−9.0)	5.6 (3.5−8.8)	6 (3.7−9.2)	0.98 (0.95−1.00)	0.185

Data are represented as mean (standard deviation), absolute (relative) frequencies or median (IQR: interquartile range); Odds Ratio (OR), 95% confidence interval and *p* value obtained via logistic regression with cluster robust standard errors. Abbreviations: BMI—body mass index; IPA—tobacco pack-years; mMRC—modified Medical Research Council; %predicted post-BD FEV1—percent predicted post-bronchodilator FEV1; GesEPOC—Spanish National Guidelines for COPD; Triple therapy—long-acting beta-2 agonists, long-acting antimuscarinic agents and inhaled corticosteroids.

**Table 3:** Multivariate logistic regression to identify independent factors associated with testing of levels of AAT in COPD patients.

Variable	OR (95%CI)	*p*
Genotyping of AATD availability		
Not (reference)	1	
Yes	0.42 (0.26−0.68)	0.001
Attended in specialized COPD outpatient clinic		
Not (reference)	1	
Yes	1.88 (1.19−2.99)	0.007
%FEV1 postBD		
≥80% (reference)	1	
50−79%	1.50 (1.08−2.08)	0.013
<50%	1.86 (1.36−2.55)	<0.001
BMI kg/m^2^		
>21 (reference)	1	
≤21	1.18 (0.91−1.53)	0.193
Age (years)		
>55 (reference)	1	
≤55	1.84 (1.18−2.85)	0.007
Gender		
Male (reference)	1	
Female	1.16 (0.97−1.40)	0.101
Number of hospital admissions in the last year		
<1 (reference)	1	
≥1	1.25 (0.96−1.64)	0.093
Charlson index		
<3 (reference)	1	
≥3	0.63 (0.52−0.76)	<0.001
History of asthma		
Not (reference)	1	
Yes	1.53 (0.96−1.43)	0.069

Odds Ratio (OR), 95% confidence interval and *p* value obtained via logistic regression with cluster robust standard errors. Abbreviations: BMI—body mass index; %predicted post-BD FEV1—percent predicted post-bronchodilator FEV1.

**Table 4:** Multilevel logistic regression models of the variations in testing AAT between the 2 audits.

Number of Participating Hospitals	Total Number of Patients Assessed in the Two Audits	% Patients with Levels of Alpha-1 Antitrypsin Serum Testing in Audit 2015	% Patients with Levels of Alpha-1 Antitrypsin Serum Testing in Audit 2021	OR (95 % CI)	*p*	Adjusted OR * (95 % CI)	*p*
25	4573	408 (18.9)	934 (38.7)	2.70 (1.70−4.28)	<0.001	2.81 (1.63−4.84)	<0.001
Center and patient characteristics in the 2015 and 2021 audits of variables of interest for AAT testing in COPD							
				In Audit 2015 (*n* = 2157)	In Audit 2021 (*n* = 2416)		*p*
Genotyping of AATD availability, *n* (%)				1566 (72.6)	2406 (99.6)		<0.001
Number of attendees in specialized COPD outpatient clinic, *n* (%)				765 (35.5)	1300 (53.9)		<0.001
%FEV1 postBD < 50%, *n* (%)				658 (44.6)	1196 (49.6)		0.011
BMI (kg/m^2^) ≤ 21, *n* (%)				156 (7.3)	229 (10)		0.001
Age (years) ≤ 55, *n* (%)				184 (8.5)	138 (5.7)		<0.001
Female, *n* (%)				318 (14.7)	685 (28.4)		<0.001
Number of hospital admissions in the last year ≥ 1, *n* (%)				538 (24.9)	380 (15.7)		<0.001
Charlson index ≥ 3, *n* (%)				920 (42.7)	666 (27.6)		<0.001
Have history or symptoms of asthma, *n* (%)				540 (25)	237 (9.8)		<0.001
Chronic bronchitis criteria, *n* (%)				843 (39.1)	661 (27.4)		<0.001
Home ventilation, *n* (%)				177 (8.2)	224 (9.3)		0.111

* The adjustment variables of the model were adjusted by being treated at a specialized COPD outpatient clinic, age ≤ 55 years old, FEV1 < 50%, BMI ≤ 21, female, number of hospital admissions in the last year ≥ 1, medical history of asthma, home ventilation, Charlson index ≥ 3 and genotyping of AATD availability. Odds Ratio (OR), 95% confidence interval and *p* value obtained via logistic regression with cluster robust standard errors.

**Table 5:** Characteristics of patients and procedures conducted during the follow-up for COPD evaluation according to AAT levels (binary logistic regression).

Characteristics of the Patient Tested for AAT *n* = 1651	Patients with AAT Level < 60 mg/dL *n* = 66 (4%)	Patients with AAT Level ≥ 60 and <116 mg/dL *n* = 265 (16%)	Patients with AAT Level ≥ 116 mg/dL *n* = 1320 (80%)	OR (95%CI) AAT Level ≥ 60 and <116 mg/dL vs. ≥116 mg/dL (Reference)	*p*	OR (95%CI) AAT Level < 60 mg/dL vs. ≥116 mg/dL (Reference)	*p*
AAT plasma level (mg/dL), m(SD)	19.3 (14.6)	98.9 (14.2)	160.9 (58.0)				
Clinical characteristics							
Age (years), m (SD)				0.98 (0.96−0.99)		0.99 (0.92−1.06)	0.836
>55, (n, %) (reference)	67.6 (11.4)	66.7 (8.5)	68.1 (8.8)	1	0.015	1	
≤55, (n, %)	12 (18.2)	23 (8.7)	114 (8.6)	1.00 (0.61−1.63)	0.985	2.34 (0.89−6.15)	0.082
Sex (female), (reference)				1		1	
Male n (%)	49 (74.2)	187 (70.6)	919 (69.6)	1.04 (0.77−1.41)	0.772	1.25 (0.61−2.58)	0.533
Active smokers, n (%)							
No (reference)				1			
Yes	12 (18.2)	58 (21.9)	364 (27.6)	0.73 (0.45−1.18)	0.206	0.58 (0.36−0.92)	0.023
IPA (Pack-years), m (SD)	42.7 (19.9)	47.7 (24)	49.4 (23.1)	0.99 (0.98−1.00)	0.454	0.98 (0.96−1.00)	0.192
BMI kg/m^2^, m (SD)	26.4 (4.0)	27.5 (5.2)	27.1 (5.8)	0.6 (0.98−1.03)	0.426	0.97 (0.93−1.01)	0.174
>21 n (%) (reference)				1		1	
≤21 n (%)	5 (7.7)	23 (9.1)	144 (11.2)	0.79 (0.48−1.28)	0.347	0.66 (0.18−2.42)	0.536
Dyspnea (MRC-m), n (%)							
<2 (reference)				1		1	
≥2	46 (75.4)	109 (51.4%)	584 (54.4)	0.88 (0.64−1.21)	0.457	2.56 (1.67−3.94)	<0.001
Charlson index, n (%)							
<3 (reference)				1			
≥3	24 (36.4)	45 (17)	315 (23.9)	0.65 (0.46−0.91)	0.012	1.81 (0.99−3.33)	0.053
Chronic bronchitis criteria, n (%)							
Not (reference)				1			
Yes	27 (40.9)	78 (29.4)	439 (33.3)	0.83 (0.61−1.14)	0.260	1.38 (0.52−3.69)	0.510
History of asthma, n (%)							
Not (reference)				1			
Yes	3 (4.5)	35 (13.6)	167 (12.8)	1.07 (0.67−1.68)	0.768	0.32 (0.06−1.65)	0.176
GesEPOC Phenotype, n (%)							
Non-exacerbator (reference)	18 (33.3)	89 (48.4)	404 (43)	1		1	
Exacerbator chronic bronchitis	21 (38.9)	25 (13.6)	141 (15)	0.80 (0.42−1.52)	0.504	3.34 (1.21−9.20)	0.020
Exacerbator with emphysema	12 (22.2)	36 (19.6)	228 (24.3)	0.71 (0.45−1.11)	0.143	1.18 (0.66−2.09)	0.560
Asthma-COPD	3 (5.6)	34 (18.5)	166 (17.7)	0.92 (0.53−1.62)	0.798	0.40 (0.13−1.18)	0.098
Number of hospital admissions in the last year, n (%)							
<1 (reference)				1		1	
≥1	25 (37.9)	46 (17.4)	240 (18.2)	0.94 (0.65−1.35)	0.761	2.74 (0.84−8.87)	0.092
FEV1 (%), m (SD)	52.8 (16.8)	51.5 (17.6)	50.4 (18.4)	0.6 (0.99−1.00)	0.335	1.00 (0.99−1.02)	0.411
≥80, n (%) (reference)	31 (47)	14 (5.3)	78 (5.9)	1		1	
50–79%, n (%)	31 (47)	125 (47.2)	562 (42.6)	1.23 (0.63−2.41)	0.914	1.07 (0.24−4.78)	0.924
<50%, n (%)	4 (6.1)	126 (47.5)	679 (51.5)	1.03 (0.56−1.89)	0.528	0.89 (0.33−2.33)	0.813
BODE index, m (DE)	3.8 (2.2)	3.1 (2.2)	4.0 (2.0)	0.83 (0.74−0.93)	0.003	0.95 (0.76−1.19)	0.689
Long-term oxygen therapy, n (%)							
Not (reference)				1		1	
Yes	18 (27.3)	56 (21.1)	329 (24.9)	0.80 (0.60−1.07)	0.149	1.12 (0.53−2.36)	0.747
Diagnostic procedures conducted during the follow-up for COPD evaluation							
Diffusion capacity measured on any occasion, n (%)							
Not (reference)				1		1	
Yes	51 (77.3)	265 (80.1)	996 (75.5)	1.36 (0.94−1.97)	0.100	1.10 (0.56−2.16)	0.769
Lung volumes measured on any occasion, n (%)							
Not (reference)				1		1	
Yes	21 (31.8)	790 (58.8)	970 (58.8)	1.00 (0.72−1.39)	0.970	0.31 (0.09−1.00)	0.051
6-min walk test carried out on any occasion, n (%)							
Not (reference)				1		1	
Yes	27 (40.9)	786 (59.5)	962 (58.3)	0.87 (0.62−1.22)	0.431	0.47 (0.09−2.34)	0.358
BODE index calculated on any occasion, n (%)							
Not (reference)				1		1	
Yes	17 (25.8)	565 (42.8)	699 (42.3)	1.05 (0.77−1.44)	0.733	0.46 (0.08−2.63)	0.386
Chest CT carried on any occasion, n (%)							
Not (reference)				1		1	
Yes	65 (98.5)	1091 (82.7)	1381 (83.6)	1.18 (0.85−1.63)	0.320	13.6 (1.36−136.0)	0.026

Data are represented as mean (standard deviation) or absolute (relative) frequencies or median (IQR: interquartile range); Abbreviations: AAT—alpha-1 antitrypsin; IPÂ—tobacco pack-years; BMI—body mass index; mMRC—modified Medical Research Council; %predicted post-BD FEV1—percent predicted post-bronchodilator FEV1; GesEPOC—Spanish National Guidelines for COPD; BODE—body mass index, airflow obstruction, dyspnea and exercise capacity; 6MWT—6-min walk test; CT—computerized tomography; Odds Ratio (OR), 95% confidence interval and *p* value obtained via logistic regression with cluster robust standard errors.

## Data Availability

The data presented in this study are available on request from the corresponding author.

## Supplementary Materials

The following supporting information can be downloaded at: https://www.mdpi.com/article/10.3390/jcm13040955/s1, Table S1: Participants in EPOCONSUL study; Table S2: The inclusion criteria and exclusion criteria; Table S3: Hospital-related and patient-related variables.
