# Supplementary material for: Detection of Alpha-1 Antitrypsin Levels in Chronic Obstructive Pulmonary Disease in Respiratory Clinics in Spain: Results of the EPOCONSUL 2021 Audit
Source: J Clin Med. 2024 Feb 7;13(4):955. doi: 10.3390/jcm13040955 (PMC10889736; doi:10.3390/jcm13040955)
Supplement: Supplementary file 1 [file jcm-13-00955-s001.zip › jcm-2844848-supplementary.pdf]

SUPPLEMENT

SUPPLEMENT Table S1: Participants in EPOCONSUL study.

Región	Researchers and centres
Andalucía	José Calvo Bonachera. H. Torrecárdenas. Almería, Virginia Almadana Pacheco. H. U Virgen de la Macarena. Málaga, Francisco Marín Sánchez. H. U. Virgen de la Victoria. Málaga, J. L López Campos. H. U Virgen del Rocío. Sevilla.
Aragón:	María Angeles Gótor Lazaro. Hospital Universitario Miguel Servet. Ana Boldova. Zaragoza, Hospital Royo Villanova. Zaragoza, Virginia Mo, Cristina Aguilar Paesa. H. Clínico. Zaragoza.
Asturias	Marta Iscar Urrutia, Ana Pando Sandoval, Cristina Hernández González. Hospital Universitario Central de Asturias. Oviedo,
Murcia	María Jesús Avilés Inglés. Hospital General. Universitario Reina Sofía, Juan Miguel Sánchez Nieto. Hospital Morales Meseguer, Mª Carmen Fernández Sánchez. H. Universitario Rafael Méndez. Murcia
Canarias	Marco Acosta Sorense. Hospital Universitario Nuestra Señora de la Candelaria. Tenerife.
Cantabria	Beatriz Abascal. H. U de Valdecilla. Santander.
Castilla y la Mancha	José Alfonso García Guerra. H. Mancha Centro. Alcazar de San Juan. Ciudad Real.
Castilla y León	Ana Pueyo. Hospital Universitario de Burgos, José Luis Fernández Sanchez, María Bartol Sanchez, Tamara Clavero Sanchez, Laura Gil Pintor. H. U. de Salamanca.
Cataluña	Miriam Barrecheguren. H Val de Hebrón. Barcelona, Noelia Pablos Mateos. H. Sant Joan de Déu de Martorell. Barcelona, Sandra Marin. Hospital Dos de Maig de Barcelona, Annie Navarro. Hospital U Mútua de Terrassa. Barcelona, Elena de Miguel Campos. Hospital Moisés Broggi Sant Joan Despí. Barcelona.
Valencia	José María Tordera. Hospital Universitario La Fe de Valencia, Dolores Martínez Pitarch. Hospital LLuis Alcanys de Xátiva. Valencia, Marta Palop Cervera. Hospital de Sagunto. Valencia, Lia Alonso Tomás, Marta Solé Delgado. H Arnau de Vilanova. Valencia, Cruz Gonzalez Villaescusa. Hospital Clínico Universitario de Valencia, Eusebi Chiner Vives. H. U. San Juan. Alicante.
Extremadura	Francisca Lourdes Marquez, Luis Miguel Sierra Murillo. Hospital Universitario de Badajoz, Juan Antonio Riesco. H. San Pedro de Alcantara. Mirian Torres González. Hospital Virgen del Puerto de Plasencia
Baleares	Francisco Fanjul Losa. Hospital Universitario Son Espases. Palma de Mallorca, Antonia Fuster Gomila. Hospital Universitario Son Llatzer.

	Palma de Mallorca.
Madrid	Soledad Alonso Viteri. H de Torrejon. Madrid, Aurora Solier. H Ramón y Cajal. Madrid, Andrea Yordi. H. Infanta Elena. Valdemoro. Madrid, Nuria Arenas, Blas Rojo. Hospital Infanta Sofía San Sebastián de los Reyes. Madrid, Juan Luis Rodriguez Hermosa, Gianna Vargas Centanaro. H. Clínico San Carlos. Madrid, Manuel Valle Falcones. H. U Puerta de Hierro Majadahonda. Madrid, Tamara Alonso Perez, Rosa Mar Gómez Punter, Elena García Castillo. H. La Princesa. Madrid, J. De Miguel, Zichen Ji. H. U. Gregorio Marañón. Madrid, Carolina María Gotera Rivera. Fundación Jimenez Diaz. Madrid.
Navarra	José Espinoza Pérez. Complejo Hospitalario de Navarra.
País Vasco	María Milagros Iriberri Pascual, Patricia Sobradillo Ecenarro. H de Cruces. Vizcaya, Raquel Sánchez Juez. Hospital Universitario de Basurto. Vizcaya, Cristobal Esteban González. Hospital de Galdakao. Vizcaya.

SUPPLEMENT Table S2. The inclusion criteria and exclusion criteria.

The inclusion criteria	- patients aged ≥40 years<br>- smokers or ex-smokers (of at least 10 pack-years)<br>- COPD diagnosed on the basis of spirometric tests (FEV1/FVC post-bronchodilation<0.7 or FEV1/FVC pre-bronchodilation<0.7 and FEV1≥80%, if there is no bronchodilation reversibility testing available
The exclusion criteria	- lack of follow-up for at least 1 year in a respiratory outpatient clinic<br>- participating in a clinical trial

SUPPLEMENT Table S3. Hospital-related and patient-related variables.

Hospital-related variables

- Autonomous Community of Spain
- Province where the hospital belongs
- Population assigned for admission to the hospital
- Level of hospital: primary-level I: has few specialities, mainly internal medicine, obstetrics-gynecology, pediatrics, and general surgery; limited laboratory services are available for general but not for specialized pathological analysis; bed capacity ranges from 30-200 beds. Secondary-level II: highly differentiated by function with five to ten clinical specialities and bed capacity from 200-800 beds; often referred to as provincial hospital. Tertiary-level III: highly specialized staff and technical equipmen, ICU and specialized imaging units; clinical services are highly differentiated by function; bed capacity from 300-1500 beds; often referred to as central, regional or tertiary-level hospital.Is it a university hospital?
- Is there inpatient clinic in the Respiratory unit?
- Respiratory hospitalization beds
- Are there Pulmonology residents in the hospital?
- Total number of Pulmonology residents in the beginning of the study
- Number of staff pulmonologists in the Unit (excluding residents)
- Which of the following specialized respiratory outpatient clinics are available in your Respiratory unit?
- How many minutes do you usually have for a scheduled first time visit in a specialized respiratory outpatient clinic?
- How many minutes do you usually have for a scheduled follow-up visit in a specialized respiratory outpatient clinic?
- How many minutes do you usually have for a scheduled first-time visit in the general respiratory outpatient clinic?
- How many minutes do you usually have for a scheduled follow-up visit in the general respiratory outpatient clinic?
- Is there a nursing respiratory in the outpatient clinic?
- How many nursing respiratory outpatient clinics are there?
- Does your Respiratory unit have a specialized COPD respiratory outpatient clinic?
- How many scheduled visits are there in a week in the specialized COPD respiratory outpatient clinic?
- Does the specialized COPD respiratory outpatient clinic have a nurse available?
- What is the nurse profile?
- Does the Unit have an organized educational program on inhaler use for the outpatient COPD patients?
- Who runs it?
- Does the center have forced spirometry available?
- Does the center have static lung volumes measurement available?
- Does the center have diffusing capacity measurement available?
- Does the center have respiratory muscle strength measurement available?
- Does the center have sputum eosinophil count available?
- Indicate how often you use it in your clinical practice
- Does the center have sputum culture available?
- Does the center have cardiorespiratory exercise testing available?
- Does the center have echocardiography available?
- Does the center have simple thoracic X-ray available?
- Does the center have chest CT scan available?
- Does the center have total serum IgE determination available?
- Does the center have serum Alfa-1-antitrypsin determination available?
- Does the center have genetic testing for Alfa-1-antitrypsin deficiency available?

– Does the center have 6MWT available?<br><br>– Does the center have CAT questionnaire available in the outpatient clinic?<br>– Does the center have a respiratory rehabilitation program available?<br>– Of what type:<br>– Is there any type of protocoled nutritional support for COPD patients?
**Patient-related variables**<br>– Follow-up start and final date<br>– Visit type<br>– Patient gender<br>– Patient age<br>– Years of clinical follow-up<br>– Smoking history available<br>– Number of cigarettes per day<br>– Number of years smoking<br>– Last spirometry testing<br>– Post-bronchodilation spirometry - Date<br>– Post-bronchodilation spirometry - FEV1 (ml)<br>– Post-bronchodilation spirometry - FEV1 (%)<br>– Post-bronchodilation spirometry - FVC (ml)<br>– Post-bronchodilation spirometry - FVC (%)<br>– Post-bronchodilation spirometry - FEV1/FVC<br>– Does the patient participate in any clinical trials or research projects?<br>– Does the patient meet all the inclusion criteria?<br>– What Unit referred the patient to the Respiratory outpatient clinic?<br>– What is the type of outpatient clinic that follows the case?<br>– Date of current visit<br>– Date of previous scheduled follow-up visit<br>– What is the smoker status of the patient?<br>– Patient's weight in the last spirometry report (Kg)<br>– Patient's height in the last spirometry report(m)<br>– Are the comorbidities defined in the clinical record, including?<br>– Cardiopathy<br>– Peripheral arterial disease<br>– Arterial hypertension<br>– Dyslipidemia<br>– Neurologic disease<br>– Sleep apnea<br>– Depression<br>– Anxiety<br>– Chronic respiratory disease<br>– Of what type:<br>– Other respiratory diseases<br>– Asthma<br>– Rhinitis<br>– Digestive disorders<br>– Metabolic syndrome<br>– Diabetes<br>– Chronic renal disease<br>– Neoplastic disease<br>– AIDS<br>– Connective tissue diseases<br>– Bone, joint and muscle disorders<br>– Total Charlson index

- Bronchodilator reversibility testing
- % reversibility
- Is serum Alfa-1-antitrypsin determination available?
- Measured value
- Is sputum culture performed on any occasion?
- Cardiopulmonary exercise testing performed on any occasion?
- Is Chest CT scan carried out on any occasion?
- Reason for performing chest CT scan?
- Is 6MWT performed on any occasion?
- Date of 6MWT
- Distance recorded
- Are Symptoms or quality of life questionnaires determined on any occasion?
- Date CAT
- Score CAT
- Is Lung volume measurement performed on any occasion?
- Is diffusion capacity performed on any occasion?
- Are Arterial blood gases performed on any occasion?
- BODE index calculated on any occasion?
- Value BODE
- Is the BODEx index calculated on any occasion?
- Value BODEx
- Dyspnea grade according to mMRC
- Does the patient currently meet the clinical criteria for chronic cough and/or chronic expectoration?
- Is there chronic colonization by any microorganism?
- Is the number of moderate/severe exacerbations in the last 12 months recorded during the visit?
- Number
- Review clinical record and indicate number
- Is the number of hospital admissions in the last 12 months recorded?
- Number
- Review clinical record and indicate number
- Is smoking history recorded during the visit?
- Is exercise routine recorded during the visit?
- Is annual influenza vaccination recorded during the visit?
- Is pneumococcal vaccination recorded during the visit?
- Specify if any complementary examination is considered during the current visit
- Bronchodilation reversibility testing
- Lung volumes
- Diffusing capacity
- Arterial blood gases
- 6MWT
- Total serum IgE
- ReasonEchocardiography
- CT
- Symptoms questionnaires
- Is the current COPD medication reported in the clinical record?
- Which of the following pharmacologic treatments is the patient prescribed:
- Is treatment adherence evaluated in some way?
- Is the grade of satisfaction with the inhaler devise recorded?
- Is inhalation technique evaluated in some way?
- Was the pharmacologic treatment modified compared with the previously used?
- Indicate any changes
- Long-term oxygen therapy

- Home mechanical ventilation
  - Does the patient participate in a respiratory rehabilitation program?
  - Is a specific intervention for smoking cessation offered?
  - Indicate
  - Is exercise recommended during the visit?
  - Is vaccination recommended during the visit?
  - Indicate
  - Is COPD severity defined in the clinical record?
  - Indicate
  - Is the GesEPOC phenotype defined in the clinical record?
  - Indicate
  - Is the next scheduled visit indicated in the clinical record?
  - Next follow-up visit in. (months)
  - Was the patient discharged from the clinic?
